# Impact of 24-week dapagliflozin treatment on body weight, body composition, and cardiac risk indicators of patients with type-2 diabetes mellitus

**DOI:** 10.55730/1300-0144.5683

**Published:** 2023-08-11

**Authors:** Pınar AKHANLI, Sema HEPŞEN, İsmail Emre ARSLAN, Hakan DÜĞER, Hayri BOSTAN, Muhammed KIZILGÜL, Bekir UÇAN, Erman ÇAKAL

**Affiliations:** Division of Endocrinology and Metabolism, Department of Internal Medicine, Dışkapı Yıldırım Beyazıt Training and Research Hospital, Ankara, Turkiye

**Keywords:** SGLT-2 inhibitors, dapagliflozin, body weight, fat mass, carotid intima-media thickness

## Abstract

**Background/aim:**

To reveal the impacts of dapagliflozin, a sodium glucose transporter-2 inhibitor (SGLT-2i), on body weight and body composition, cardiovascular risk indices, and carotid intima-media thickness (CIMT).

**Materials and methods:**

The data of patients with type-2 diabetes mellitus (T2DM) who applied to Department of Endocrinology and Metabolic Disorders between September 2019 and 2020, and had started dapagliflozin treatment along with their current medications were recorded retrospectively. Body weights, body compositions measured through bioelectrical impedance, and CIMT with T2DM receiving SGLT-2i treatment and medication were measured at weeks 1, 12, and 24 of 42. The visceral adiposity index (VAI), lipid accumulation product (LAP), and atherogenic index of plasma (AIP) were used to determine the lipid measurements and anthropometric values.

**Results:**

The mean change in the total body weight and total fat mass was −2.96 and −1.97 kg, respectively (p < 0.001). There was a reduction in total fat mass of 1.23 kg (from 31.4 to 29.3 kg, p < 0.001) and in body fat percentage of 2.5% (from 35.8% to 34.4%, p < 0.001) in the first 12 weeks. A mild increase was observed in both the total fat mass and body fat percentage between weeks 12 and 24, which was not statistically significant (p = 0.783 and p = 0.925, respectively), whereas there was a statistically significant reduction in high-sensitive C-reactive protein (hsCRP), AIP, and CIMT values (p = 0.006, p = 0.035, and p = 0.007, respectively). No changes were observed in the VAI and LAP values (p = 0.985 and p = 0.636, respectively).

**Conclusion:**

It was observed that dapagliflozin not only contributes to weight and fat loss but also has positive impacts on cardiovascular and atherosclerotic indicators.

## 1. Introduction

Type-2 diabetes mellitus (T2DM) is an important risk factor for cardiovascular morbidity and mortality [1]. It also accompanies obesity, which is the other cardiovascular risk factor. For this reason, ensuring weight loss and reaching glycemic targets in T2DM treatment is also important. Some treatment methods used for T2DM, such as sulfonylurea, thiazolidinedione, and insulin, can cause an increase in weight and visceral fat tissue [2].

Sodium glucose transporter-2 inhibitors (SGLT-2i) are medications used to ensure blood glucose regulation by preventing glucose reabsorption from the kidney proximal tubule. Large-scale clinical studies have revealed that SGLT-2i have unprecedented cardiorenal impacts in patients with T2DM and a cardiovascular disease (CVD) or multicardiovascular risk factors. SGLT-2i not only have antidiabetic and cardioprotective impacts, but also cause weight loss [3]. Weight loss may be derived from either the loss of calories depending upon urinary glucose excretion or fluid loss secondary to osmotic diuresis or the combination of both factors [4]. Ensuring weight loss in T2DM treatment is a substantial strategy in terms of both the prognosis of the disease and patient motivation [5]. It is a known fact that dapagliflozin improves the cardiovascular outcomes by decreasing blood pressure, body weight, fat mass, triglyceride, and uric acid levels in addition to blood glucose normalization [6].

Various parameters and indices are used in order to predict CVD and atherosclerosis risk, including the carotid intima-media thickness (CIMT), high-sensitive C-reactive protein (hsCRP), lipid accumulation product (LAP), atherogenic index of plasma (AIP), and visceral adiposity index (VAI) [7–10]. The aim of this study was to evaluate the impacts of 24-week dapagliflozin treatment on weight loss, body fat distribution measured through bioelectrical impedance (BIA), and cardiovascular indicators. The improvement of CVD-atherosclerosis was assessed using cardiovascular risk indicators such as the CIMT, VAI, AIP, and LAP.

## 2. Material and methods

### 2.1. Study design

This research was designed as a retrospective observational study. Ethical approval was received from our institute in accordance with the principles of the Declaration of Helsinki. Before participation in this study, written informed consent was given by all of the participants.

### 2.2. Patients, laboratory tests, and anthropometric measurements

Patients with T2DM who applied to the Department of Endocrinology and Metabolic Disorders between September 2019 and 2020, and began dapagliflozin treatment along with their current medications were recorded retrospectively. The BIA and CIMT values of the patients who began SGLT-2i treatment were also measured. During the treatment, physical examination records of the patients at baseline, and weeks 12 and 24 were reviewed. The patients included in the study were being treated with oral antidiabetic agents, only insulin, or oral antidiabetic agents, except for SGLT-2 along with insulin. Patients whose treatment regime had been changed within the last 12 weeks or whose treatment included an agent that caused their weight to change were excluded from the study. The study population comprised 75 patients. The medication was discontinued in 1 and 5 patients due to euglycemic diabetic ketoacidosis and urinary tract infection and/or genital candidiasis, respectively. On the other hand, 27 patients could not be included in the study because they did not attend the 3- and 6-month follow-up. The height (cm), weight (kg) (barefoot and in thin clothes), body mass index (BMI) (kg/m^2^), waist circumference (WC) (with a flexible measuring tape, at the level of the umbilicus, and standing), and hip circumference (HC) (with a flexible measuring tape, at the widest circumference over the gluteus maximus and the symphysis pubis) of the patients were measured. A standard hypocaloric diet and regular aerobic exercise were suggested to all of the T2DM patients.

Body composition was measured using the BIA (Tanita DC 360, Tokio, Japan). The measurement was carried out barefoot and with thin clothing, without any metallic materials. All of the blood samples were drawn from the antecubital vein at all of the visits, following a 12-h fast and without taking any medication.

A noninvasive high-resolution ultrasound of the common carotid artery with a 13-MHz linear probe (Hitachi, Japan; EUB 7000) was used in order to estimate the CIMT value. The distance between the blood-intima and media-adventitia boundaries was used to define the CIMT, and the CIMT value was determined by calculating the mean value of consecutive 3 measurements. Measurements were carried out on B-mode imaging from a distance of 1 cm to the internal carotid arterial bifurcation, at which the hemodynamics were only minimally affected. All of the measurements were carried out by the same researcher (P.A.).

Cardiovascular indices were calculated using the formulas given below:


$$\begin{array}{l} \text{LAP}=(\text{WC }[\text{cm}]-65)\times (\text{TG }[\text{mmol}/\text{L}])\;(\text{for the males})\\ \text{LAP}=(\text{WC }[\text{cm}]-58)\times (\text{TG }[\text{mmol}/\text{L}])\;(\text{for the females}) \end{array}$$[
8]

$$\begin{array}{l} \text{VAI}=(\text{WC }[\text{cm}]/(39.68+(1.88\times \text{BMI})))\times (\text{TG}/1.03)\times (1.31/\text{HDL-C }[\text{mmol}/\text{L}])\;(\text{for the males})\\ \text{VAI}=(\text{WC }[\text{cm}]/(36.58+(1.89\times \text{BMI})))\times (\text{TG}/0.81)\times (1.52/\text{HDL-C }[\text{mmol}/\text{L}])\;(\text{for the females}) \end{array}$$[
10]

AIP=log TGs/HDL-C[
9]

### 2.3. Statistical analysis

IBM SPSS Statistics for Windows 21.0 (IBM Corp., Armonk, NY, USA) was used for the statistical analysis. In order to determine if the variables were normally distributed, analytic (Shapiro-Wilk test) and visual methods (histograms and probability plots) were used. The nonparametric measurements at 3 time points (baseline, 3 months, and 6 months) were compared through the Friedman test. Comparison of the repeated measurements of the parametric variables was done using 1-way analysis of variance (ANOVA) test. A post hoc analysis was conducted when p < 0.05. A 5% type-I error was used to infer statistical significance. Means and standard deviation for normally distributed variables and medians and interquartile ranges (IQRs) for nonnormally distributed variables were used in order to present the descriptive analyses. P < 0.05 was considered statistically significant. Correlations among the variables and their significance were investigated using the Spearman test.

## 3. Results

Included in the study were 42 patients, of whom 18 were male (IQR: 25–75, 42.9) and 24 were female (IQR: 25–75, 57.1). The median age of the patients was 53 years (IQR: 25–75, 45–71). DM-induced complications, comorbid status, antidiabetic medications, and the current smoking status of the patients are presented in Table 1.

The mean change in the total body weight (TBW) was −2.96 kg (p < 0.001). There was a 2.4-kg reduction (from 84.75 to 80.75 kg) between weeks 0 and 12, and a 1.4-kg reduction (from 80.75 to 79.1 kg) between weeks 12 and 24. When comparing the results at baseline with those of week 12, there was a significant reduction in the TBW, BMI, WC, and HC (p < 0.001), which continued in the following weeks, but not to the same extent as in the first 12 weeks. The mean change in the total fat mass was −1.97 kg (p < 0.001). There was also a 1.23-kg reduction in the total fat mass between weeks 0 and 12 (from 31.4 to 29.3 kg) (p < 0.001). Similarly, a 2.5% reduction (from 35.8% to 34.4%) was observed in the body fat percentage during the same timeframe (p < 0.001). A mild increase, which was not statistically significant, was also observed in both the total fat mass and body fat percentage between weeks 12 and 24 (p = 0.783 and p = 0.925, respectively).

The hemoglobin A1c (HbA1c), fasting plasma glucose (FPG), and postprandial glucose (PPG) values decreased between weeks 0 and 12 (p **<** 0.001, p = 0.001, and p = 0.002, respectively). This reduction continued to decrease between weeks 12 and 24 (p = 0.146, p = 0.111, p = 0.132, respectively). No significant changes were observed in the creatinine levels.

There were also no changes in the total cholesterol, low-density lipoprotein (LDL) cholesterol, and triglyceride levels. On the other hand, there was an increase (p = 0.002) in the high-density lipoprotein (HDL) cholesterol (p = 0.002) levels between weeks 0 and 12, but there was no change between weeks 12 and 24.

The cardiovascular indicators values between weeks 0 and 24 were also evaluated. There was a statistically significant reduction in the hsCRP, AIP, and CIMT values (p = 0.006, p = 0.035, and p = 0.007, respectively), whereas there was no change in VAI and LAP values (p = 0.985 and p = 0.636) According to the results of the Friedman test, there was a significant difference in both the systolic and diastolic blood pressures between 0 and 24 weeks (p **<** 0.001, p = 0.032, respectively) (Table 2).

The reduction in the WC of the patients and weight loss was positively associated with the reduction in the LAP values (r = 0.391, p = 0.01; r = 0.389, p = 0.011). There was a weak positive correlation among the reductions in the BMI and VAI, LAP, and AIP (r = 0.338, p = 0.029; r = 0.414, p = 0.06; r = 0.343, p = 0.026, respectively). Moreover, the reduction between the body fat percentage and LAP and AIP was positively correlated (r = 0.335, p = 0.03; r = 0.335, p =0.03, respectively). No correlation was observed among the hsCRP and CIMT, and body composition and glucose metabolism parameters.

## 4. Discussion

Herein, it was determined that dapagliflozin caused a weight loss in the 24-week follow-up period. The rate of weight was explicit, particularly in the first 12 weeks and it was observed with the reduction in the total fat mass and body fat percentage. Contrary to other studies on SGLT-2i, an increase in total fat mass and body fat percentage was detected despite the fact that there was no reduction in weight at the follow-ups after week 12. However, adding dapagliflozin to the current treatment was associated with a reduction in cardiovascular risk factors like the hsCRP, CIMT, and PAI.

It is known that SGLT-2i cause weight loss [11]. Bolinder et al. revealed that dapagliflozin caused a 2.96-kg reduction in 24 weeks in patients taking metformin, similar to the current study [4]. Many patients in our sample group had been using insulin (73.8%). The weight loss detected in the current study was similar to the published reports related to insulin and SGLTi. Dapagliflozin, at a dose of 10 mg, caused a 2.43- and 3.33-kg reduction within 48 and 104 weeks, respectively, in patients using insulin compared to the placebo [12]. While ipragliflozin caused a 2.78-kg reduction within 24 weeks, canagliflozin, at a dose of 100 and 300 mg, caused a 1.9- and 2.4-kg reduction, respectively, within 18 weeks compared to the placebo [13, 14]. When evaluated only in the first 3 months, the mean change in the TBW was similar to that of tofogliflozin and luseogliflozin [15,16]. The pattern of change in the TBW continued to increase during first several weeks and then decreased. Even though most of our patients were overweight or obese and their eating habits were different from those in other countries, their weight loss and weight loss patterns had similar characteristics as the other studies conducted in the countries mentioned above.

At the end of the study, a reduction in both the total fat mass and body fat percentage was observed compared to baseline. The total fat mass was reduced by 1.97 kg in 24 weeks. In the follow-ups after week 12, there was a reduction in the TBW, whereas there was an increase in the body fat mass and body fat percentage. A study in which the body fat mass was measured through BIA revealed that the total fat mass was reduced by 2.21 kg with ipragliflozin treatment [13]. Studies in which the change in the fat mass was evaluated via dual X-ray absorptiometry revealed that the total fat mass had decreased gradually by week 24 with both dapagliflozin and ipragliflozin treatments [13,17]. It was also revealed in a long-term study that dapagliflozin had caused a 1.34-kg reduction in the total fat mass during a 102-week follow-up and this reduction continued until week 102 [17]. It was opined that this was the result of the patients doing less physical activity as a consequence of the COVID-19 pandemic, which began during our follow-ups.

It was considered that the weight loss as a result of SGLT-2i was due to both osmotic diuresis secondary to glycosuria and the loss of calories [18]. SGLT-2i increase glyconeogenesis, lipolysis, and glucagon levels [19]. Glycosuria caused by SGLT-2i decreases plasma glucose and insulin levels. Fasting and postprandial glucagon concentrations are increased by SGLT-2i. A reduction in glucose levels in the circulation with these changes results in the mobilization of lipid storage. Lipolysis increases in adipose tissue and nonesterified fatty acids transformed into ketone bodies in the liver are released [20]. These mechanisms support the opinion that the loss of calories secondary to glycosuria, as a result of SGLT-2i, is responsible for weight loss. In addition to this, it should be taken into consideration that fluid loss may have contributed to a rapid decrease in TBW [4].

When the weeks between 0 and 24 were evaluated, there were no changes observed in the VAI and LAP values and no decrease was seen in the hsCRP, AIP, and CIMT values. It was observed that the reductions in the BMI were weakly positively correlated with the VAI, LAP, and AIP values.

The CIMT is accepted as an indicator of CVDs in patients with T2DM. Moreover, DM leads to the progression of the CIMT [21]. As best as could be determined, there are no studies in literature that have evaluated the short-term impact of dapagliflozin treatment on the CIMT and whether it causes any changes in the AIP and LAP. A 104-week study in which tofogliflozin treatment was compared to conservative DM treatment revealed that the mean CIMT and right-left maximum CIMT decreased in both study groups compared to the initial values. Nevertheless, there was no significant difference in CIMT progression between the tofogliflozin and conservative treatment groups [22]. In another study, it was reported that no statistically significant difference was observed in the CIMT after a 52-week ipragliflozin treatment in 134 Japanese patients with T2DM [23]. However, it was determined herein that dapagliflozin had a significant impact on the CIMT, even within a short time period of 24 weeks.

The AIP is an important indicator of atherosclerosis and coronary heart disease [24]. In a study conducted with diabetic patients, the AIP was correlated with the body weight, BMI, WC, fasting plasma glucose, postprandial blood glucose, HbA1c, and homeostasis model assessment of insulin resistance [25]. The AIP decreased after a 24-week dapagliflozin treatment. There was also a correlation between the AIP and BMI and body fat percentage. Herin, no significant changes were observed in VAI and LAP values. It was also reported that empagliflozin substantially decreases the VAI value [26]. The differences in the results of the current study may have resulted from the limited number of patients or perhaps the fact that the COVID-19 pandemic emerged during the study period, as indicated before. Moreover, that fact that there was an increase in body fat mass in the follow-ups after week 12 may have been the other reason that there was no decrease in the VAI and LAP values. In our opinion, on the other hand, that the change in the hsCRP, CIMT, and AIP values may have been the result of the impact of SGLT-2i on the inflammatory markers.

However, the reasons why SGLT-2i contributed to an improvement in cardiovascular indicators were not clearly revealed. It was opined that it was effective by decreasing the inflammatory markers caused by atherosclerosis in addition to blood glucose regulation. In a study conducted using animal models, lipid accumulation, intimal proliferation, and all of the proinflammatory marker levels were suppressed with SGLT-2i treatment, which indicates that this medication has a beneficial antiatherosclerotic effect. These analyses revealed that SGLT-2i treatment substantially reduces not only macrophage infiltration but also the suppression of inflammatory M1 markers, including tumor necrosis factor-alpha, interlukerin-1 (IL-1), and IL-6, which play a significant role in the improvement of atherosclerosis. Moreover, the SGLT-2i treatment did not affect the blood lipid profile, which led us to believe that it may have a direct therapeutic effect on preventing atherosclerosis [6].

There were some limitations to the current study. Due to the COVID-19 pandemic, some of the patients did not come back to the hospital, despite the fact that they should have been followed-up at certain times. For this reason, the number of patients was limited. Moreover, no objective or quantitative data were collected about the food and fluid intake of the patients. Additionally, since the sample size was limited, it was not possible to analyze gender-related differences.

The fact that SGLT-2i ensures weight and fat loss, and has several positive impacts on DM is very crucial in preventing other complications of obesity and increasing the motivation of patients. It was revealed that dapagliflozin treatment contributes to an improvement in cardiovascular risk factors such as CIMT, hsCRP, and PAI, in addition to its common positive cardiac outcomes. These finds are important because, as we have observed, the COVID-19 pandemic has caused additional challenges in the glucose regulation and weight management of diabetic patients.

## Figures and Tables

**Table 1:** Demographics, comorbidities, complication rates, and details of the DM treatments of the patients.

Number (n)	42
Age (years)	53 (45–71)
Duration of DM (year)	15 (1–35)
Sex	
Male, n (%)	18 (42.9)
Female	24 (57.1)
Micro-vascular complications	
Diabetic nephropathy	8 (19)
Diabetic neuropathy	20 (47.6)
Diabetic retinopathy	9 (21.4)
Comorbidities	
Hypertension	18 (42.9)
Coronary artery disease	8 (19)
Dyslipidemia	17 (40.5)
Cerebrovascular disease	1 (2.4)
Use of other antidiabetic medications	
Sulfonylurea	5 (11.9)
Metformin	39 (92.9)
DPP-4 inhibitor	17 (40.7)
Insulin	31 (73.8)
Current smoking status	9 (21.4)

DPP-4: Dipeptidyl peptidase-4. Age and duration of DM are presented as the median and IQR 25–75. Other descriptive parameters are presented as n (%).

**Table 2:** Impact of dapagliflozin treatment on body composition, cardiovascular risk indices, and laboratory parameters.

	Baseline	12 weeks	Change from baseline–12 weeks	CI	24 weeks	Change from baseline 12–24 weeks	CI	p-value
Body composition parameters								
Body weight (kg)	84.75 (73.75, 95.25)	80.75 (71.5, 92.5)	−2.4 (−4.5, −1.3)	−3.8, −1.95	79.1 (69.4, 93.3)	−0.3 (−1.8, 1.27)	−1.4, 0.78	**<0.001**
BMI (kg/m^2^)	33.43 ± 5.8	32.29 ± 6	−1.13 ± 1.23	0.75, 1.51	32.35 ± 6.3	0.1 (−0.62, 0.65)	−0.58, 0.29	**<0.001**
WC (cm)	107.7 ± 12.9	104.9 ± 13	−2.85 ± 3.1	1.87, 3.83	104.6 ± 13.7	−0.24 ± 3.1	−0.7, 1.2	**<0.001**
HC (cm)	116.9 ± 11.5	114.6 ± 11.9	−2.24 ± 2.92	1.33, 3.14	114 (103.75, 124.25)	−0.6 ± 2.2	−0.09, 1.3	**<0.001**
Total fat mass (kg)	31.4 ± 10.4	29.3 ± 10.35	−2 ± 2.7	1.23, 2.94	29.4 ± 10.7	0.11 ± 2.7	−0.95, 0.72	**<0.001**
Body fat percentage (%)	35.8 ± 8.7	34.4 ± 8.9	−1.2 (−2.4, −0.1)	−2.5, −0.7	34.7 ± 9.1	0.15 (−1.6, 1.4)	−0.74, 0.44	**<0.001**
Cardiovascular risk indices								
VAI	2.52 (1.68, 4)	2.23 (1.5, 3.75)	−0.17 (−0.74, 0.26)	−0.43, 0.04	2.26 (1.4, 3.7)	0.03 (−0.55, 0.56)	−0.21, 0.36	0.218
AIP	0.19 ± 0.26	0.13 ± 0.29	−0.06 (−0.16, 0.07)	−0.09, 0.01	0.13 ± 0.31	−0.001 ± 0.2	−0.6, 0.9	0.097
LAP	84.7 (52.4, 114.9)	74.19 (46.5, 111)	−4.2 (−26.2, 7.9)	−12.2, 4.75	71.8 (40.4, 109.8)	−2.52 (−15, 20.3)	−8.2, 13.6	0.377
hsCRP	4.2 (2.6, 7.6)	4 (1.4, 6)	−0.4 (−1.1, 2)	−1, 2.6	2.7 (1.7, 4.6)	−1 (−2.7, 0.2)	−2.7, 0.2	0.066
CIMT (mm)	0.74 ± 1.35	–	–	–	0.7 ± 1.25	−0.04 ± 0.1	0.01, 0.07	**0.007**
Systolic blood pressure (mm/Hg)	120 (120, 130)	120 (113, 120)	−5 (−10, 0)	−0.10 −0.25	110 (110, 120)	−2.5 (−10, 0)	−0.5, 0	**<0.001**
Diastolic blood pressure (mm/Hg)	80 (70, 80)	75 (70, 80)	−5 (−10, 0)	−10, 0	70 (70, 72)	0 (−6.25, 0)	−5, 0	**0.032**
Laboratory parameters								
Hba1c (%)	9.3 (8.4, 10.5)	8.6 (7.4, 9.6)	−1.1 (−1.6, −0.3)	−1.5, −0.6	8.3 ± 1.15	−0.2 (−0.5, 0.3)	−0.3, 0.18	**<0.001**
Fasting plasma glucose (mg/dL)	211 (153, 257)	163 (125, 231)	−43.6 ± 81.1	18.3, 68.9	143 (120, 181)	−7.5 (−49, 14)	−27, 11	**<0.001**
Postprandial glucose (mg/dL)	301.6 ± 83.7	254 ± 79	−47.6 ± 95.2	17.9, 77.3	237.2 ± 72.3	−16.8 ± 70.9	−5.3, 38.9	**0.001**
Creatinine	0.85 ± 0.17	0.83 ± 0.16	−0.01 (−0.08, 0.08)	−0.06, 0.01	0.82 (0.66, 0.98)	0.02 ± 0.11	−0.05, 0.15	0.445
ALT	22 (16, 35)	20 (17, 31)	−3 (−7, 0.5)	−6.4, −1	19 (12, 26)	−1.7 ± 7.8	−1.2, 4.6	**0.02**
Total cholesterol (mg/dL)	193 ± 49	186 (152, 230)	1 (−9, 16)	−4.4, 13.5	187 ± 45.8	−5 (−16, 7)	−13, 4.9	0.723
LDL cholesterol (mg/dL)	123 ± 40.6	128.7 ± 34	−0.5 (−10, 11)	−5.7, 3.3	122.5 ± 36.8	−5.5 (−16, 5)	−14.6, 2.6	0.413
HDL cholesterol (mg/dL)	45 ± 9.5	48 ± 10.6	2 (−0.25, 5.25)	0.05, 4.8	49 ± 10.7	1 ± 5.35	−2.6, 0.68	**0.002**
Triglyceride (mg/dL)	158 (101, 225)	147 (101, 209)	−3.5 (−35.7, 25)	−30, 5	153 (98, 228)	2.5 (−38.7, 35.5)	−19.7, 36.8	0.81

hsCRP: High-sensitive C-reactive protein, CIMT: carotid intima-media thickness, LDL: low-density lipoprotein, HDL: high-density lipoprotein, CI: confidence interval. Normally distributed variables are presented as the mean ± SD, and non-normally distributed variables are presented as median (IQR: 25–75). P-values were detected using Friedman test for nonparametric and 1-way ANOVA test for parametric variables.
